# Development of criteria-led discharge for patients undergoing abdominal wall reconstruction

**DOI:** 10.1007/s10029-025-03556-9

**Published:** 2026-01-06

**Authors:** Abdulaziz Elemosho, Benjamin A. Sarac, Vijay Raj, Vimal Narula, Daniel S. Eiferman, B. Todd Heniford, Jeffrey E. Janis

**Affiliations:** 1 Department of Plastic and Reconstructive Surgery, College of Medicine, The Ohio State University Wexner Medical Center, 915 Olentangy River Road, Suite 2100, Columbus, OH 43212 USA; 2 Department of Surgery, The Ohio State University Wexner Medical Center, Columbus, OH USA; 3 Division of Gastrointestinal and General Surgery, Department of Surgery, Endeavor Health, Evanston, IL USA

**Keywords:** Discharge criteria, Discharge checklist, Abdominal wall reconstruction, Hernia repair, ERAS protocol, Patient safety

## Abstract

**Background:**

Despite the wide adoption of Enhanced Recovery After Surgery (ERAS) protocols, no formal discharge criteria have been validated for patients undergoing abdominal wall reconstruction (AWR). This study evaluates the validity and utility of a 15-item discharge checklist following AWR.

**Methods:**

We retrospectively reviewed 128 consecutive adult patients who underwent elective open AWR by a single surgeon between 2021 and 2023, and a 15-item binary checklist was applied. Criteria validity was assessed by concordance with discharge decisions, while utility was evaluated using 30-day complication rates and hospital length of stay (LOS). Multivariate logistic regression was conducted before and after propensity adjusting for age, patient comorbidities and procedure complexities.

**Results:**

Of 128 patients, 67 (52.3%) met all 15 checklist criteria on the day of discharge, while 61 (47.7%) did not. Those who met full criteria had significantly shorter LOS (4.0 vs. 5.8 days; *p* < 0.001), lower 30-day complication rates (15.6% vs. 52.6%; *p* = 0.006) and a lower rate of 30-day readmission (1.5% vs. 11.5%, *p* = 0.027) compared to those who did not. The checklist had a sensitivity of 80.9% and specificity of 56.3% for predicting uneventful discharge. Meeting all 15 checklist criteria was associated with lower odds of 30-day complication requiring intervention [adjusted odd ratio aOR) 0.12 (0.04–0.41) *p* < 0.001 and 30-day readmission [aOR 0.12 (0.02–0.79) *P* = 0.027].

**Conclusions:**

A structured 15-item checklist is clinically valid, accurately identifies patients safe for discharge after open AWR, can reduce LOS, and predict postoperative complications. These findings support checklist-guided, criteria-led discharge as a valuable adjunct to ERAS protocols in AWR.

**Supplementary Information:**

The online version contains supplementary material available at 10.1007/s10029-025-03556-9.

## Introduction

Patients undergoing abdominal wall reconstruction (AWR) may face prolonged hospitalizations due to the complex nature of their comorbidities, operative course, and postoperative management [1, 2]. In other surgical subspecialties, such as bariatric and colorectal surgery, structured discharge checklists have helped standardize patient readiness and reduce variability of timing of discharge without increasing readmissions [2, 3]. These tools, which often incorporate vital signs, laboratory thresholds (e.g., hemoglobin, white blood cell count), pain control, ambulation status, and oral intake, have never been validated for the AWR population, one of the most complex surgical cohorts in elective surgery [4–7].

While Enhanced Recovery After Surgery (ERAS) programs have standardized many elements of perioperative care, such as early ambulation and transversus abdominis plane (TAP) blocks, these interventions are primarily aimed at improving recovery, not at defining discharge readiness [8, 9]. Data from Carolinas Medical Center (CMC) suggest that even five years after ERAS implementation, LOS in open AWR patients did not significantly differ compared to the pre-ERAS era (6.5 vs. 7.2 days, ) [10], underscoring the need for alternative strategies tailored to discharge decision-making.

AWR patients often represent a high-risk subpopulation of patients, requiring complex, multidisciplinary procedures with variable postoperative recovery. In this context, we believe that discharge decisions made without objective criteria may contribute to unnecessary prolongation of hospitalization, especially in academic settings where care transitions and weekend cross-coverage are common. Prolonged hospitalization is independently associated with hospital-acquired complications, increased costs, and worse patient outcomes [11, 12].

This study evaluates the validity and utility of a 15-item discharge readiness checklist specific to AWR that could provide a reproducible, objective framework for safely identifying patients who are appropriate for discharge. Beyond optimizing care, a criteria-led discharge model has the potential to improve outcomes, namely, reduce length of stay (LOS) and postoperative complications.

## Methods

Following Institutional Review Board (IRB) approval, we designed a retrospective cohort study to evaluate the validity and utility of a 15-item discharge readiness checklist among patients undergoing AWR. The study included all adult patients who underwent elective AWR by the senior author between 2021 and 2023 at a single academic tertiary care center. Patients were included if they were between 18 and 99 of age and had complete postoperative documentation, including all daily post-operative documentation and post-op clinic visits. Patients who had approaches other than open repair or had concomitant plastic surgery procedures were excluded.

### Checklist design and application

Our team adopted a 15-item binary discharge checklist that was developed at CMC by a team of surgeons and research fellow based on prior literature and institutional experience [3, 13]. It spanned four domains: “history” (willingness for discharge, IV fluid bolus, pain control and social support), physical exam (vital signs, drain and urine output), laboratory safety parameters (hemoglobin value, white blood cell count and renal function) and surgeon’s evaluation (Surgeon assessment of readiness for discharge) Table 1. Each day, the research team reviewed the patients’ chart, including labs, vitals, and progress notes, and determined which of these criteria were met and which were not. The purpose of having the “Surgeon Evaluation section” is to routinely identify barriers to discharge that are not encompassed by the discharge checklist and to use attending experience in the subtle areas of patient evaluation.

**Table 1:** Discharge criteria checklist

CATEGORY	MEASUREMENT	PARAMETER
History	Patient Willingness Hemodynamics Pain Social	Patient willing to go home No fluid bolus over last 24 h Pain able to be controlled by oral pain medications Patient has appropriate place for discharge (home, Skilled Nursing Facility (SNF) etc.)
Physical Exam	Abdominal Exam Temperature Heart Rate (HR) Systolic Blood Pressure (SBP) Oxygen Saturation Urine Output Drain Content	No peritoneal signs; no fascial dehiscence; no hematoma Temp 101.4 F HR 55–100 bpm SBP 100–170 O2 Sat > 90% (If history of COPD, > 88%) on room air. If on home oxygen, must be on home setting > 0.5 cc/kilogram/hour Serosanguinous or serous output in drain
Lab Findings	Hemoglobin White Blood Cell count (WBC) Creatinine (Cr)	**≤** 2 gram drop in Hgb WBC within normal limits (3–11.5) Cr ≤ 1.5x baseline
Surgeon Evaluation	Subjective Evaluation	Surgeon feels, based on subjective evaluation, that the patient is ready for discharge **Reason**,** if not**: _______________

The checklist was applied on the day of discharge, and each item was marked as “Met” or “Not Met.” Patients who met all 15 items were considered to have met the full checklist criteria. For secondary analyses, we also assessed patients meeting 14 of 15 criteria. Fulfillment of each individual item and their predictive value for discharge readiness were recorded and analyzed.

### Assessment of validity

Checklist validity was defined as the ability of the tool to align with the actual clinical decision to discharge the patient. To evaluate this, we calculated the proportion of patients who were discharged despite not meeting the full checklist, and conversely, the proportion who met the checklist and were successfully discharged. This also represents the positive predictive value (PPV) of the checklist for discharge readiness.

### Assessment of utility

Checklist utility was assessed in terms of safe discharge and length of stay (LOS). Safe discharge was defined as absence of 30-day surgical site occurrences (SSO) (defined as any infection, seroma, hematoma, dehiscence, enterocutaneous fistulas or skin necrosis) [14] requiring unplanned reintervention. To test the predictive utility of the checklist for safe discharge, we compared the 30-day SSO requiring reintervention and 30-day readmission rates between those who met full criteria and those who did not. Sensitivity and specificity were determined for the checklist in its utilization for safe discharge.

### Data collection and variables

Demographic, clinical, operative, and postoperative outcome data were extracted from the electronic medical record and cross-verified for accuracy. Variables collected included age, sex, body mass index (BMI), American Society of Anesthesiologists Physical Status American Society of Anesthesiologists Physical Status (ASA) physical status classification, ventral hernia working group (VHWG) grade, defect size (cm²), operative time, comorbidities, discharge disposition, and 15 checklist items.

### Statistical analysis

Continuous variables were reported as means and standard deviations; categorical variables were reported as counts and percentages. Between-group comparisons were made using Welch’s t-tests for continuous variables and Chi-square or Fisher’s exact tests for categorical variables. A two-sided p-value < 0.05 was considered statistically significant. Checklist performance characteristics (sensitivity, specificity, and PPV) were calculated using standard formulas. Individual checklist items were also analyzed for their standalone PPV. 30-day complications requiring reintervention and 30-day readmission were modeled using logistic regression with a binary exposure indicating meeting all 15 criteria checklist or not, adjusting for age, BMI, ASA ≥ 3, defect size, operating time, and concomitant plastic procedure.

For propensity matching, a single propensity score (PS) was estimated from the above covariates (to avoid over-adjustment given limited events). As a co-primary secondary analysis, we evaluated dose–response to determine the incremental effect of meeting each of the criterion. Firth logistic regression was used only if separation occurred.

## Results

### Criteria validity

#### How do the criteria conform with the decision to discharge patients?

A total of 128 patients met inclusion criteria. Of these, 67 (52.3%) met all 15 checklist criteria, while 61 (47.7%) did not (check-list negative). Notably 118 patients, (92.2%) met at least 14 of the 15 criteria before discharge.

The hemoglobin criterion was the most common reason for not meeting the full criteria.

#### How do the criteria conform to patients’ complexity?

Checklist-negative patients were significantly older (57.4 vs. 62.4 years, *p* = 0.023), had a higher BMI (31.7 vs. 33.5 kg/m², *p* = 0.022), larger defects (129.2 vs. 166.8 cm², *p* = 0.014) And were more likely to have ASA ≥ 3 status (77.0% vs. 52.2%, *p* = 0.008). Other baseline demographic data are as shown in Table 2.

**Table 2:** Patients demographics

Variable	Met (*n* = 67)	Not Met (*n* = 61)	*p*-value
Mean Age **	57.4	62.4	0.002
Mean BMI **	31.7	33.5	0.035
BMI > 30 kg/m² *	41 (61.2%)	49 (80.3%)	0.247
Diabetes *	16 (23.9%)	20 (32.8%)	0.537
Hypertension *	41 (61.2%)	36 (59.0%)	0.336
COPD *	4 (6.0%)	6 (9.8%)	0.532
Immunosuppression *	8 (11.9%)	7 (11.5%)	0.826
Primary Hernia Diagnosis *	28 (41.8%)	16 (26.2%)	0.042
Prior Mesh *	31 (46.3%)	41 (67.2%)	0.122
Closed Incisional Wound Vac *	49 (73.1%)	53 (86.9%)	0.712
Primary Fascial Closure *	51 (76.1%)	43 (70.5%)	0.072
Unilateral Component Separation *	7 (10.4%)	3 (4.9%)	0.198
Bilateral Component Separation *	31 (46.3%)	42 (68.9%)	0.094
Bridged Repair	0 (0.0%)	0 (0.0%)	—
Concomitant Plastic Surgery	9 (13.4%)	2 (3.3%)	
Prior Hernia Repair (0)	20 (29.9%)	15 (24.6%)	—
Prior Hernia Repair (1)	18 (26.9%)	11 (18.0%)	—
Prior Hernia Repair (2)	11 (16.4%)	15 (24.6%)	—
Prior Hernia Repair (≥ 3)	7 (10.4%)	20 (32.8%)	—
Mean Defect Width (cm) **	9.4	11.3	0.017
Mean Defect Length (cm) **	12.1	14.4	0.015
Mean Defect Size (cm²) **	129.2	166.8	0.014
Total OR Time (min) **	321.7	355.4	0.109
ASA Status = 1	3 (4.5%)	0 (0.0%)	—
ASA Status = 2	29 (43.3%)	14 (23.0%)	—
ASA Status ≥ 3	35 (52.2%)	47 (77.0%)	—

**t-test

*chi square test

### Criteria utility

#### Discharge readiness

The positive predictive value (PPV) of the individual checklist item for discharge readiness is assessed under four domains (Table 3):

**Table 3:** Proportion of discharge criteria met

Parameter	Criterion Positive Predictive Value for Discharge
Patient willing to go home	100.0% (*n* = 128)
No fluid bolus over the last 24 h	100.0% (*n* = 128)
Pain is able to be controlled by oral pain medications	100.0% (*n* = 128)
Patient has appropriate place for discharge (home, SNF, etc.)	100.0% (*n* = 128)
No peritoneal signs; no fascial dehiscence; no hematoma	100.0% (*n* = 128)
Temp 101.4 F	100.0% (*n* = 128)
HR 55–100 bpm	99.2% (*n* = 127)
SBP 100–170	97.7% (*n* = 125)
O2 Sat > 90% (If history of COPD, > 88%) on room air. If on home oxygen, must be on home setting	100.0% (*n* = 128)
Urine output > 0.5 cc/kilogram/hour	96.0% (*n* = 123)
Serosanguinous or serous output in drain	100.0% (*n* = 128)
**≤** 2 gram drop in Hgb	67.2% (*n* = 86)
WBC within normal limits (3–11.5)	92.2% (*n* = 118)
Cr ≤ 1.5x baseline	97.7% (*n* = 125)
Surgeon feels, based on subjective evaluation, that the patient is ready for discharge	100% (*n* = 128)

- History/Social: Patient willing to go home (PPV 100%), no IV fluid bolus in the prior 24 h (100%), pain controlled on oral analgesics (100%), and appropriate discharge destination secured (100%). Examination/Vitals: benign abdominal exam without peritoneal signs, dehiscence, or hematoma (100%); temperature ≤ 101.4 °F (100%); heart rate 55–100 bpm (99.2%; *n* = 127); systolic blood pressure 100–170 mmHg (97.7%; *n* = 125); oxygen saturation > 90% on room air (> 88% if COPD) or patient’s home oxygen setting if applicable (100%); urine output > 0.5 cc/kilogram/hour (96.0%; *n* = 123); and serous/serosanguinous drain output (100%).
- Labs: ≤2-g hemoglobin decrease (67.2%; *n* = 86), white blood cell count within institutional limits (92.2%; *n* = 118), and creatinine ≤ 1.5× baseline (97.7%; *n* = 125).
- Surgeon assessment: attending surgeon’s readiness determination (100%).

In our overall discharge assessment of the patient cohort, the full checklist had a positive predictive value of 52.3% for discharge readiness. When the hemoglobin criterion was excluded, the predictive value rose to 80.5%.

#### Safe discharge

**Fig. 1:**
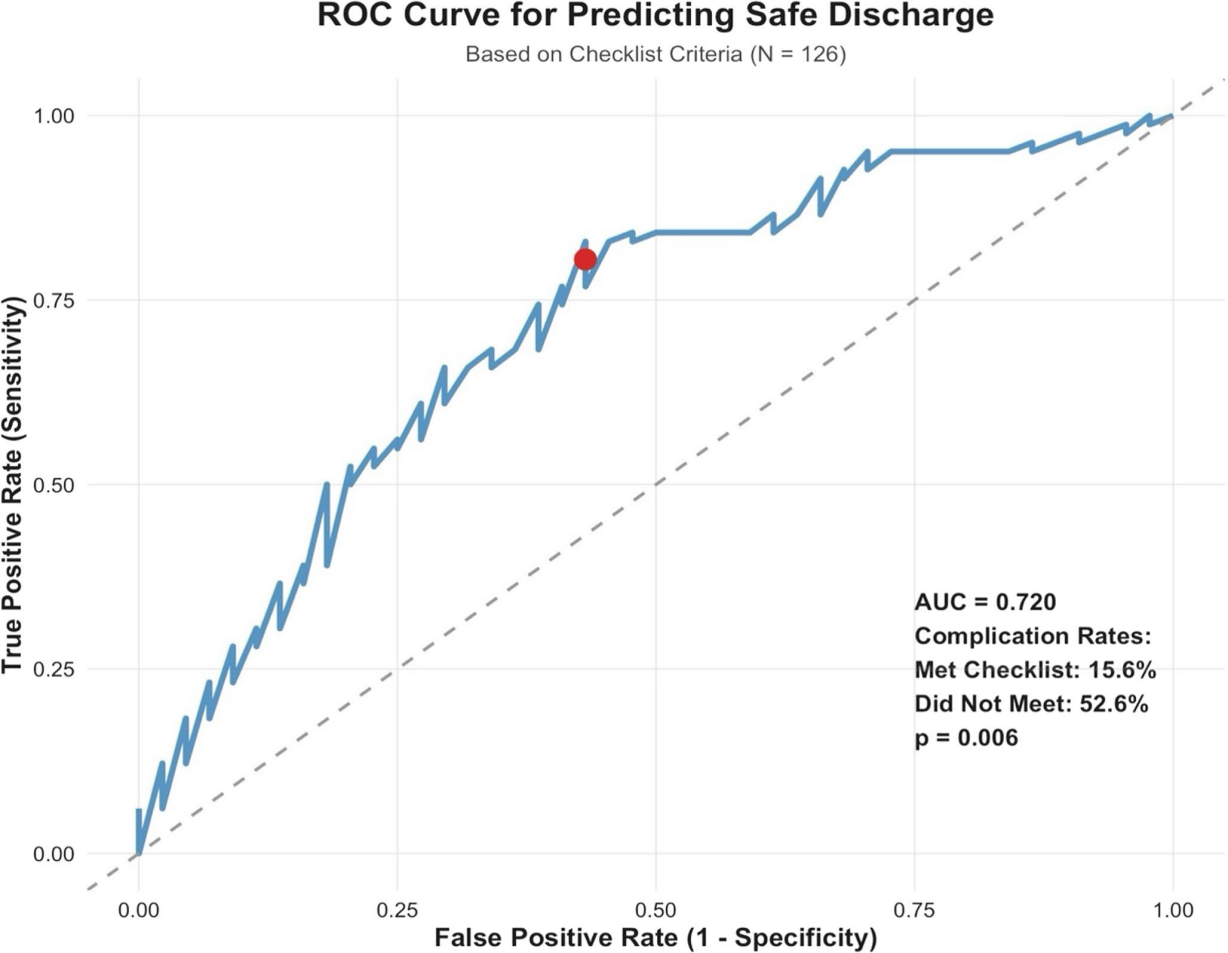
Receiver Operator Curve for Safe Discharge

Patients who met the full checklist criteria had significantly fewer 30-day complications requiring intervention compared to those who did not (14.9% [10/67] vs. 52.5% [32/61], *p* < 0.001). Meeting the full checklist showed a sensitivity of 80.9% and specificity of 56.3% for predicting a safe discharge. See Fig. 1 for the ROC curve for safe discharge. When the threshold was expanded to include patients who met ≥ 14 of 15 criteria, complication rates remained significantly lower compared to those who met < 14 items (28.8% [34/118] vs. 80.0% [8/10], *p* = 0.002), with higher sensitivity of 97.8% but reduced specificity (27.8%).

For 30-day readmissions, patients who met the full checklist criteria had lower rates than those who did not (1.5% [1/67] vs. 11.5% [7/61], *p* = 0.027). At the ≥ 14/15 threshold, 30-day readmissions were significantly lower for those who met ≥ 14 items compared to those who met < 14 items (1.7% [2/118] vs. 60.0% [6/10], *p* < 0.001).

In the full cohort analysis, meeting all 15 checklist criteria was associated with lower odds of 30-day complication requiring intervention [adjusted odd ratio aOR) 0.12 (0.04–0.41) *p* < 0.001 and 30-day readmission [aOR 0.12 (0.02–0.79) *P* = 0.027].

After propensity matching, meeting all 15 checklist criteria were associated with lower odds of 30-day complications [aOR 0.12 (0.03–0.47) *p* = 0.002]. Similar reduction was observed for readmission (aOR 0.33, 95% CI 0.04–2.66; *p* = 0.30) although not statistically significant.

Modeling the checklist as a dose-response showed significant associations: each 1-SD increase in criteria met was linked to reduced odds of complications (aOR 0.17, 95% CI 0.03–0.97; *p* = 0.0496) and readmission (aOR 0.08, 95% CI 0.007–0.66; *p* = 0.023).

#### Length of stay (LOS)

Patients who met all checklist criteria had a significantly shorter LOS compared to those who did not (mean 4.0 vs. 5.8 days, *p* < 0.001).

## Discussion

This study demonstrates that a structured, 15-point discharge checklist is both valid and clinically useful in the postoperative management of patients undergoing AWR. Patients who met all the checklist criteria had a nearly 2-day reduction in LOS, a fourfold lower rate of 30-day complications requiring reintervention and a sevenfold lower rate of 30-day readmission. Even those meeting at least 14 of 15 criteria had significantly improved outcomes compared to those who did not, reinforcing its clinical value. Checklist items related to ambulation, oral intake, oxygenation, pain control, and drain output had the highest predictive value for safe discharge, while hemoglobin was the least discriminatory. These findings suggest that a criteria-led discharge approach can enhance recovery, improve outcomes, and support more predictable care pathways in complex surgical populations.

Interestingly, patients who failed to meet checklist criteria were older, had higher BMI, larger hernia defects, and ASA class ≥ 3–factors known to increase peri-operative risk [15–17]. Yet, the checklist’s ability to identify low-risk patients appropriate for discharge, despite comorbidities, offers a reproducible framework to individualize recovery trajectories.

Not all checklist components were equally valuable. Functional recovery markers such as ambulation, transition to oral intake, oxygenation, and surgeon assessment, had perfect positive predictive value for discharge readiness. In contrast, laboratory-based thresholds, especially hemoglobin drop, were less discriminative. These findings align with prior studies that caution against overreliance on lab values when functional status is acceptable [18]. Specifically, Boden et al. prospectively evaluated discharge hemoglobin in patients following abdominal surgery and found it was not independently associated with complications, readmission, or outcomes. They concluded that hemoglobin should not be used in isolation to determine discharge, particularly when functional recovery markers are met [18]. Our data support a shift in focus from arbitrary lab cutoffs to physiologic milestones.

Nevertheless, our results also reinforce existing literature on Enhanced Recovery After Surgery (ERAS) protocols, which have been shown to expedite recovery and reduce the length of stay in AWR [19–25]. Majumder et al. demonstrated that ERAS implementation shortened LOS by over 2 days and reduced readmissions from 16% to 4% in open ventral hernia repair [19]. Similarly, Jensen et al. showed that even in giant hernia repairs, ERAS protocols reduced LOS from 5.5 to 3.0 days [25]. This checklist complements the broader goals of Enhanced Recovery After Surgery (ERAS). While ERAS has standardized intraoperative and early postoperative care, discharge decisions remain highly variable. Our results extend ERAS by providing a reproducible tool to standardize the final phase of hospitalization. The checklist may be especially useful in academic centers where discharge decisions are often made by rotating teams with variable experience [26, 27]. This analysis highlights how the strict 15/15 threshold optimizes efficiency through shorter length of stay (LOS; mean 4.0 days) and lower complication rates (14.9%), but with reduced sensitivity (80.9%) for predicting safe discharge, which may result in fewer patients qualifying for early release. Conversely, the ≥ 14/15 threshold prioritizes safety by achieving higher sensitivity (97.8%), thereby identifying a broader cohort of patients suitable for uneventful discharge while maintaining significantly lower complication rates (28.8% vs. 80.0%; *p* = 0.002) compared to those meeting fewer criteria, albeit at the potential cost of modestly longer LOS and lower specificity (27.8%). To contextualize these trade-offs, we emphasize that while the ≥ 14/15 threshold may serve as a pragmatic cutoff for reducing LOS, especially in resource-constrained settings, it must be balanced against potential compromises in specificity for safe discharge. Notably, the specific unmet criterion plays a critical role; in our cohort, the hemoglobin threshold was the most frequently unmet item (67.2% positive predictive value), yet its absence did not independently correlate with higher rate of readmissions or complications. However, our dose-response modeling underscores the cumulative impact of unmet criteria: each additional unmet item incrementally reduces the checklist’s specificity for safe discharge thereby increasing the risk of compromising patient safety if multiple criteria are overlooked. Surgeons should thus tailor threshold application based on individual patient profiles, institutional priorities, and the nature of unmet items to optimize both safety and efficiency.

These findings have clear implications. For patients, a structured discharge pathway can enhance safety, reduce complications, and shorten hospital stay, even in a high-risk heterogenous surgical cohort described herein. For providers, it offers a reproducible tool that shifts decision-making from subjective assessment to validated metrics, especially important as more ventral hernia repairs migrate to outpatient or short-stay settings. For hospitals, our data suggests that embedding checklist-based discharge within ERAS improves efficiency without sacrificing safety. Finally, for policymakers and payers, these findings support criteria-led discharge as a model of value-based care for delivering shorter, safer, and more predictable recoveries. As robotic and ambulatory hernia programs expand, validated checklists like ours may serve as essential safeguards ensuring quality does not suffer in the pursuit of shorter stays.

This study has limitations. First, it is retrospective and observational, introducing possible selection bias in discharge decisions and outcome attribution. Second, while the discharge checklist was applied retrospectively and uniformly documented in the medical record, its influence as a live decision-making tool was not standardized across providers, introducing the possibility of selection or confirmation bias. Third, while restricting the cohort to open AWR improves internal validity, it may limit the applicability to minimally invasive or robotic hernia repairs. Finally, given the retrospective design of this study, the risk of attribution bias is inherent where retrospective application of the checklist may attribute outcomes to criteria fulfillment rather than underlying patient factors. Additionally, the single-surgeon, single-center design is a source of generalization bias, limiting applicability to diverse surgical practices or settings. To that end, there is a need for future prospective, interventional, multi-center studies to determine whether checklist-driven discharge protocols actively improve outcomes by reducing practice variability, rather than simply identifying patients already primed for early recovery.

## Conclusion

In summary, we present a validated 15-point discharge checklist that identifies AWR patients ready for safe, early discharge. Adherence to this tool was associated with shorter LOS and fewer complications without need for readmission, offering an objective alternative to subjective discharge decisions. Adoption of criteria-led discharge frameworks may enhance ERAS protocols, reduce hospital burden, and improve recovery for patients undergoing complex abdominal wall reconstruction.

## Supplementary Information

Below is the link to the electronic supplementary material.

Supplementary Material 1

Supplementary Material 2

Supplementary Material 3

Supplementary Material 4

Supplementary Material 5

Supplementary Material 6

Supplementary Material 7
